# Actin Dynamics as a Multiscale Integrator of Cellular Guidance Cues

**DOI:** 10.3389/fcell.2022.873567

**Published:** 2022-04-27

**Authors:** Abby L. Bull, Leonard Campanello, Matt J. Hourwitz, Qixin Yang, Min Zhao, John T. Fourkas, Wolfgang Losert

**Affiliations:** ^1^ Institute for Physical Science and Technology, University of Maryland, College Park, MD, United States; ^2^ Department of Physics, University of Maryland, College Park, MD, United States; ^3^ Department of Chemistry and Biochemistry, University of Maryland, College Park, MD, United States; ^4^ Institute for Regenerative Cures, Department of Ophthalmology and Vision Science, Department of Dermatology, School of Medicine, University of California, Davis, Davis, CA, United States

**Keywords:** actin polymerization, esotaxis, electrotaxis, actin dynamics, neutrophil, galvanotaxis, directional cell migration, multiple directional cues

## Abstract

Migrating cells must integrate multiple, competing external guidance cues. However, it is not well understood how cells prioritize among these cues. We investigate external cue integration by monitoring the response of wave-like, actin-polymerization dynamics, the driver of cell motility, to combinations of nanotopographies and electric fields in neutrophil-like cells. The electric fields provide a global guidance cue, and approximate conditions at wound sites *in vivo*. The nanotopographies have dimensions similar to those of collagen fibers, and act as a local esotactic guidance cue. We find that cells prioritize guidance cues, with electric fields dominating long-term motility by introducing a unidirectional bias in the locations at which actin waves nucleate. That bias competes successfully with the wave guidance provided by the bidirectional nanotopographies.

## Introduction

In most physiological contexts, cells experience multiple independent, and potentially conflicting, guidance signals that must be integrated to determine a direction of migration. The signaling pathways converge on, and are coupled to, the cytoskeletal machinery, most notably actin filaments that nucleate and polymerize in response to external migratory cues. Recent studies have demonstrated that the signaling pathways and the actin machinery act as an excitable system, and together can exhibit characteristic waves and oscillations (Allard and Mogilner, 2013; Devreotes et al., 2017). The feedback loops in this excitable system drive cytoskeletal rearrangements that enable cells to perform tasks such as developing integrin-mediated adhesions, forming protrusions, elongating, polarizing intracellular components, and migrating (Schoenwaelder and Burridge, 1999; Chung et al., 2001; Huang et al., 2013; Devreotes and Horwitz, 2015; Copos and Mogilner, 2020).

The excitable systems character of cells allows small variations in biochemical inputs to effect major changes in behavior. Indeed, upregulation of a single protein can be sufficient to change the characteristic length scale of actin-polymerization waves, and thereby to alter the migratory phenotype dramatically (Devreotes and Horwitz, 2015; Miao et al., 2019). However, the excitable system is modulated not only by biochemical signals (Weiner et al., 1999), but also by physical cues, including electric fields (EFs) (Zhao et al., 2006) and the texture of the local environment (Lämmermann et al., 2013). Near wound sites, immune cells, such as neutrophils, naturally experience each of the above-mentioned stimuli simultaneously, and must integrate the signals into fast, directed migration that prioritizes motion toward the wound. Chemical and electrical gradients point toward the wound, whereas collagen networks provide fibrous pathways that reflect the local microenvironment. In this work, we focus on the competition between EFs and nanotopography in guiding the actin cytoskeleton and cell motion in neutrophils. We apply a 10 V/cm EF, which is comparable in magnitude to the physiological EF range of 0.5–2 V/cm, which can prompt directed cell migration via electrotaxis (Zhao et al., 2010). EFs may further activate signaling pathways that lead to changes in the polarization of cellular components (Sun et al., 2013; Cortese et al., 2014), although prior work has offered conflicting opinions regarding the important of actin polymerization in the cellular sensing of EFs (Zhao et al., 2004; Zhao et al., 2006; Sato et al., 2009; Li et al., 2012).

We study EF guidance in competition with guidance due to nanoridges having dimensions that are comparable to those of typical extracellular matrix fibers (LeBert et al., 2018; François et al., 2021), the latter of which are ubiquitous in a typical neutrophil microenvironment (Lämmermann et al., 2013; Lämmermann and Germain, 2014). The nanoridges guide local actin dynamics in a process called esotaxis. Esotaxis leads to waves with widths on the submicron scale of the individual topographic features, and has been found to enhance the directionality and persistence of migration for a wide range of cell types (Driscoll et al., 2014; Hui et al., 2014; Sun et al., 2015; Ketchum et al., 2018; Lee et al., 2020), in a process called microthigmotaxis. Our observations of actin-wave responses in this simplified, but competitive, signaling environment provide important new insights into signal integration at the level of the excitable cytoskeletal network.

## Materials and Methods

HL-60 YFP-actin cells were a gift from the lab of Dr. Orion Weiner of the University of California, San Francisco. The cells were cultured in RPMI 1640 medium, Glutamax (Life Technologies) supplemented with 10% heat-inactivated fetal bovine serum (Gemini Bio). The cells were kept in a humidified atmosphere at 37°C and 5% CO_2_. Cells were differentiated to be neutrophil-like with a supplement of 1.3% dimethyl sulfoxide Hybri-Max (Sigma Aldrich) at 4.5 × 10^5^ cells/mL.

To create the nanotopographies, we used multiphoton absorption polymerization (Li and Fourkas, 2007). Molding techniques were employed to create many acrylic polymer replicas of the nanotopographic substrates on cover slips (Sun et al., 2018). Surfaces were coated with 10 μg/ml fibronectin (Sigma Aldrich) for 1 h.

Cells were resuspended in modified Hank’s Balanced solution (mHBSS, Sigma-Aldrich) with 1 μM N-Formyl-Met-Leu-Phe (fMLP, Sigma Aldrich), and then were then plated in a 3D-printed electrotaxis chamber composed of polylactic acid (Yang et al., 2014). Toxicity and changes in pH due to electrode byproducts were minimized by using separate baths for each AgCl electrode and the cell-mHBSS solution, and by connecting the baths using agar bridges in mHBSS (2%, 0.5 cm diameter, created with glass tubing). EF polarity switches were applied over a period of <10 s. Time-lapse imaging was performed using a Perkin Elmer spinning-disk confocal microscope with a 60× objective (1 pixel = 0.21 μm) taking images every 2 s. Data were taken on at least three different days for each condition with a total of *n* = 15, *n* = 11, and *n* = 9 cells analyzed for the flat, nanoridges parallel to the EF, and nanoridges perpendicular to the EF, respectively.

Our analysis of actin-wave dynamics was based on an optical-flow algorithm, as described previously (Lee et al., 2020). Briefly, this technique allows the flow of actin to be quantified in an unbiased manner. Optical flow uses the intensity of actin-fluorescence images to calculate spatial and temporal gradients and to determine the ground-truth intensity flow. We used the Lucas-Kanade approach with a varied weight matrix, a 4 μm × 4 μm Gaussian with a standard deviation of 0.63 μm for small-scale flow and a 12.8 μm × 12.8 μm Gaussian with a standard deviation of 2.1 μm for larger-scale flow (Lucas and Kanade, 1981).

To study the changing shape of migrating cells, we applied a snake (active-contour) algorithm, as described previously (Driscoll et al., 2012). The snake algorithm was used to extract the boundary (with 200 boundary indices) of the binarized cell with a 1:1 mapping between adjacent frames, so that one index position (50) was always oriented directly leftward. The location of the cell centroid and averaged actin intensity for each boundary index was calculated through time.

## Results

**FIGURE 1:**
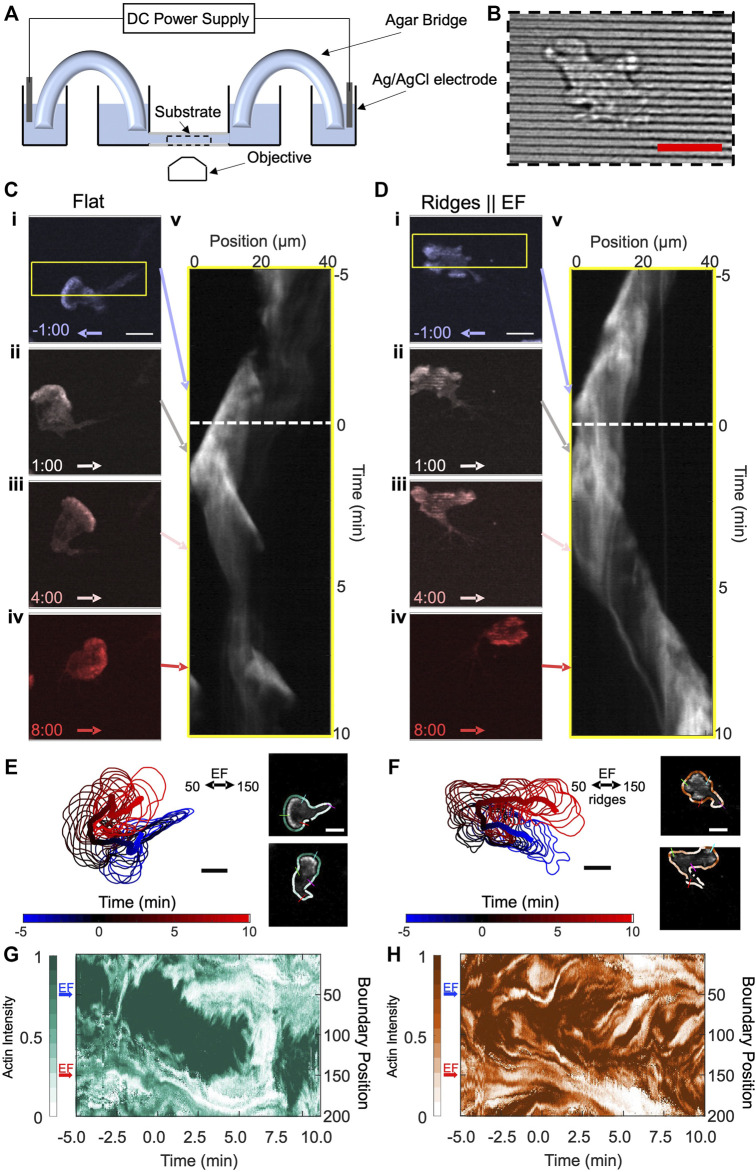
HL60 cells turn toward the new cathode within several minutes of EF reversal on both flat and nanoridged substrates, but with different local actin structures. **(A)** Schematic of the agar/salt electrotaxis chamber. **(B)** Brightfield image of resin nanoridges. **(C)** Four time-lapse snapshots of a differentiated, YFP-actin-labelled HL60 cell on a flat substrate. The cathodal (negative) direction is to the left at negative times. The EF reversal occurred at time zero. Kymograph (v) for area denoted by the yellow border in (i). **(D)** A differentiated, YFP-actin-labelled HL60 cell on nanoridges parallel to the EF. Kymograph (v) for the area denoted by the yellow border in (i). Scale bars are 10 µm. The arrows in **(C,D)** denote the EF direction, and the corresponding time slices in the actin intensity kymographs. **(E–F)** Boundary shape and path of the centroid of cell from **(C,D)** from −5:00 min to 10:00 min in 0.5-min increments, with an example of the extracted shape of a cell with labeled indices of boundary at points 50, 100, 150 and 200 on right; the boundary is colored by actin intensity. **(G)** Evolution of normalized boundary actin fluorescence intensity from **(C,E)** visualized as a kymograph. **(H)** Evolution of normalized boundary actin fluorescence intensity from **(D,F)** visualized as a kymograph. The blue and red arrows in **(G–H)** indicate the cathodal direction at the specified boundary points for negative time and positive time, respectively. Scale bars: 10 µm.

Our experiments use the neutrophil-like HL60 cell line as a well-established model for migratory immune cells (Millius and Weiner, 2010). We quantify the actin dynamics of these immune cells in response to both EFs and parallel nanoridges (see Figure 1A for experimental schematic). The nanoridges, which are spaced 1.5 μm apart, have widths (300 nm) comparable to those of collagen fibers (Figure 1B), and the EFs are comparable in magnitude to those found in wounds. For all experiments, actin-YFP tagged neutrophils are exposed to a horizontal, 10 V/cm EF. We initially align the EF from right to left (anode to cathode) and image the cells for 5 min before switching the EF direction and imaging the cell’s response for an additional 10 min. The cells are imaged on both flat resin and resin nanoridges. Figure 1C shows image stills on flat resin of the actin intensity for different time points before (i) and after (ii-iv) switching the EF. We observed a continuous 180° turn in both the actin front and cell migration direction in response to the EF (Supplementary Movie S1). The continuous turn is captured in the kymograph in Figure 1Cv. Here, the horizontal axis corresponds to the mean actin intensity in the horizontal direction (yellow box in Figure 1Ci) and the vertical axis is time. Over time, the actin intensity has a net shift to the left that eventually reverses to the right approximately 2.5 min after switching the EF (white dashed line). Interestingly, the actin front appears to persist throughout the entire experiment, and no additional protrusions are observed. Figure 1D shows results from an equivalent experiment performed on nanoridges that are aligned parallel to the EF. The actin dynamics on the ridges was less coordinated than on the flat resin, and the cell lacked a well-defined and connected actin front (Figures 1Di–iv). In Figures 1Dii–iii (Supplementary Movie S2), multiple actin patches can be seen in the image stills across the active front. However, the actin waves still exhibit a strong polarity, with the cell migration direction similar to that of the cells on flat resin. Indeed, the similar slopes of the kymographs indicate comparable speeds of the actin waves, which is consistent with our previous findings (Hui et al., 2014). The speed of the waves is roughly 4–5 μm/min and depends on a variety of factors including the polymerization rates of individual actin filaments (fast) and upstream signaling machinery (slower) (Kuhn and Pollard, 2005; Vavylonis et al., 2005; Carlsson, 2010). The primary difference between the two cases is in response to the changing EF. Instead of a smooth turning in Figure 1C, individual clusters of actin waves appeared to respond independently to the switching EF on nanoridges. Although uncoordinated, the combined responses of the actin waves drove an overall turning of the cell. The change in migration direction can be seen clearly in the kymograph in Figure 1Dv.

To capture local actin dynamics in a manner that highlights the difference between flat resin and nanoridges, we first identified and tracked the boundary of the cell. Figures 1E,F shows the time evolution of the tracked boundary and its centroid for both cells. We then parameterized the cell boundary (Driscoll et al., 2012; Driscoll et al., 2011), computed the actin concentration near each cell boundary point at each time step (Figures 1E,F right), and visualized the evolving intensity in the kymographs shown in Figures 1G,H for the flat and nanoridged substrates, respectively. In the kymographs, the vertical axis corresponds to boundary positions that start from the top of the cell (boundary position 0) and progress clockwise 360° around the entire cell to boundary position 200. For the cell on flat resin, the region of highest actin intensity initially is clearly centered in the direction of the EF (blue arrow). At time point 0, the electric field switches (red arrow). After switching, there is a continuous transition in the actin front to the new EF direction. The shift encapsulates the continuous turning of the cell that was observed in Figure 1C. Interestingly, the actin intensity reduces significantly across the entire boundary at ∼6.0 min, which we identify as a rounding-up, or cringe, period. Such behavior is commonly observed in amoeboid migration (Afonso et al., 2012). In addition, cells that were rounded up at the start of the experiment exhibited behavior consistent with activation, i.e., a transition from rounded to polarized morphology with the initial introduction of an EF on the flat substrate (Supplementary Figure S1, Supplementary Movie S3).

Figure 1H shows a kymograph of the actin intensity for the cell on nanoridges. The actin intensities along the boundary, which appear as small clusters of high intensity along the *y*-axis of the kymograph, split and merge throughout the entire experiment. During the turning of the cell, these multiple actin waves appeared to respond to the EF independently of one another. For instance, waves oriented in the anodal direction (boundary position 50) disappeared after several minutes, and waves oriented in the cathodal direction (boundary position 150) persisted. This behavior suggests that the nanoridges act as a decoupling mechanism in which the actin front observed on flat resin becomes several independent actin wave packets on nanoridges.

**FIGURE 2:**
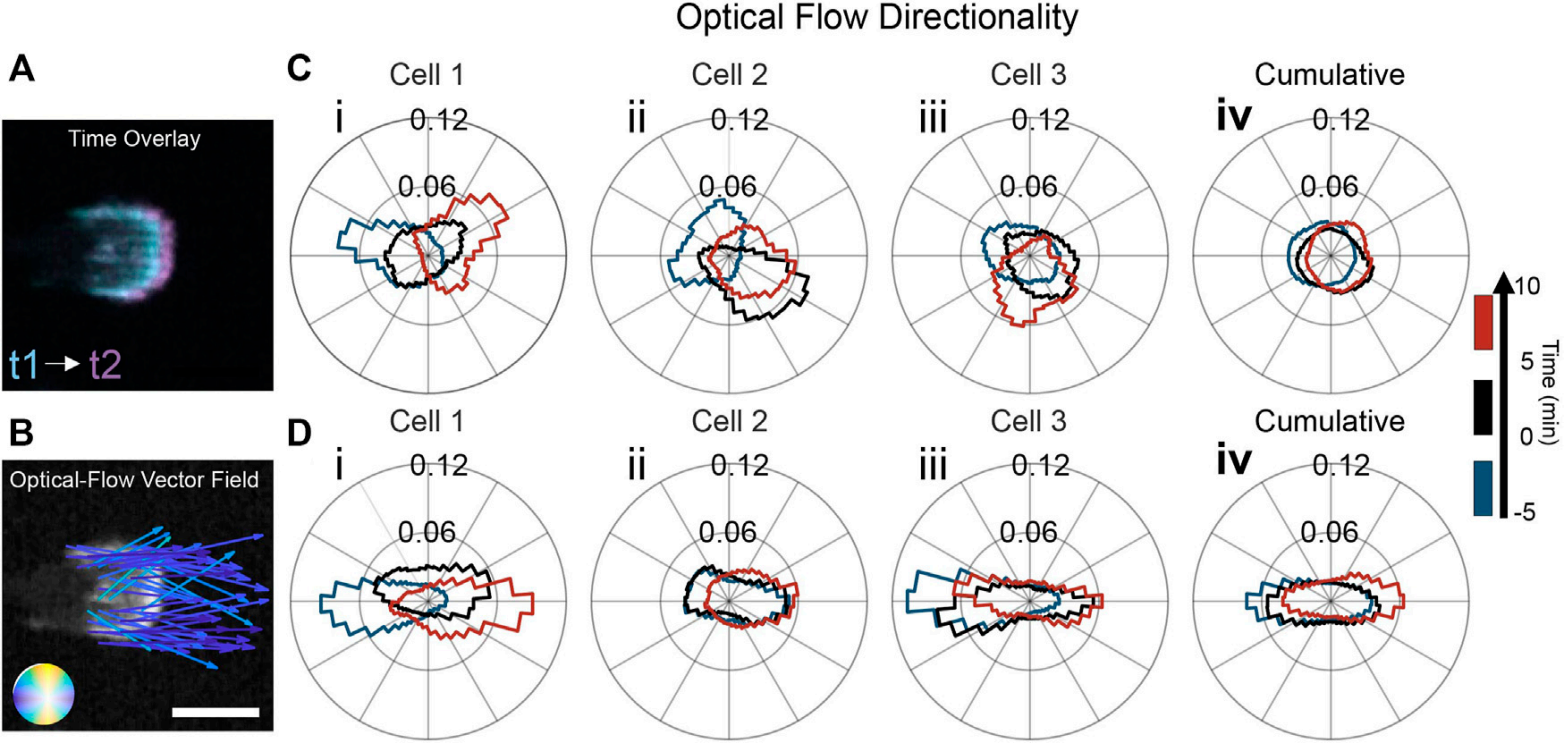
Nanoridges aligned with an EF enhances guidance of actin dynamics. **(A)** Time overlay of two actin-fluorescence images of an HL60 cell on nanoridges; the temporal spacing is 0.5 min. **(B)** Representative optical-flow vectors for the motion between the frames in **(A)**. Scale bars: 10 µm. Three characteristic examples (labeled i-iii) of individual, cell-normalized distributions of optical-flow directions on **(C)** a flat surface and **(D)** nanoridges (i from cells in Figure 1) shown for the time ranges: (blue) 5 min before the EF reversal; (black) 5 min after the EF reversal; and (red) the period from 5 to 10 min after the EF reversal. The cumulative flow-direction distribution for the same time intervals for all cells on **(Civ)** the flat surface (n = 15) and **(Div)** the nanoridges (n = 11).

To delve more deeply into the differences in actin dynamics on flat and ridged substrates, we quantified the spatial and temporal intracellular changes in an unbiased manner using optical flow (Horn and Schunck, 1981; Lucas and Kanade, 1981; Lee et al., 2020), as shown in Figure 2. Optical-flow fields use spatiotemporal actin fluorescence intensity gradients as inputs (Figure 2A), enabling quantitative characterization of the local actin-wave dynamics in the form of a spatial vector field in which each vector represents the local displacement of the actin waves in time (Figure 2B). The distribution of directions of the actin dynamics vector field for three representative cells is shown in Figure 2C. Each distribution collects data from a 5 min interval to obtain relevant statistics. The distributions reveal that the preferred direction of actin dynamics follows the cathode direction; when the electric field switches, the preferred orientation switches as well. Figure 2Ci shows the distribution of actin-wave orientations for the cell in Figure 1C. The actin flow changed its direction of motion from the left to the right, with an intermediate turning period in which the flow was oriented equally left and right. Interestingly, for all cells on flat resin, the EF changed the actin flow direction by ∼180°. However, in each experiment, there was substantial variability in the polarity cells, with ∼25% of cells polarized and migrating towards the anode before the switch of EF direction. For example, the cell in Figure 2Cii experienced actin flow directed at a mean angle of 150° before the switch. The switch in EF direction then caused the actin waves to orient towards 330°. In Figure 2Ciii, the cell underwent a 180° flip in the flow between the first and second time intervals (blue to black). In the third time interval, the flow has lost its left/right bias and is oriented perpendicular to the EF. There are two main variability factors in the actin response. First, some cells already have a preferred actin flow direction due to cell polarity when an EF is first applied. Second, the time the actin flow takes to respond to the switch in EF varies considerably among experiments. Flow reorientation (measured from cumulative distributions of flow direction over 5-min sliding windows) occurs on average when 4 min of the sliding window are after the EF switch but can take more than twice as long (Supplementary Figure S3). Due to this variability, the cumulative distribution (Figure 2Civ) is nearly isotropic.

The flow distributions for all the experiments using nanoridges were biased more strongly along the EF axis than were the distributions on flat substrates. The alignment of actin waves is evident in the three experiments presented in Figures 2Di–iii, as the flow distributions are more elongated than the distributions on flat substrates. In Figure 2Di, the flow distribution for the cell in Figure 1D is quantified. Compared to the flat substrate, there is stronger guidance of the actin waves in the direction of the EF on nanoridges. In Figure 2Dii, the flow was initially bidirectional and then shifted to the right in the third time interval. The actin flow in the final experiment (Figure 2Diii) was guided unidirectionally by the EF before the field reversal (blue). After the field switch, the flow continued in the new anode direction (black), before becoming bidirectional along the nanoridges in the third time interval (red). The nanoridges restrict the polarity to the horizontal direction, thus allowing for stronger coupling of actin waves when the EF is parallel to the ridges. When actin flow in cells of all polarity is analyzed together, the distribution of actin flow directions is nearly isotropic on flat surfaces (Figure 2Civ), but is elongated on nanoridges (Figure 2Div).

**FIGURE 3:**
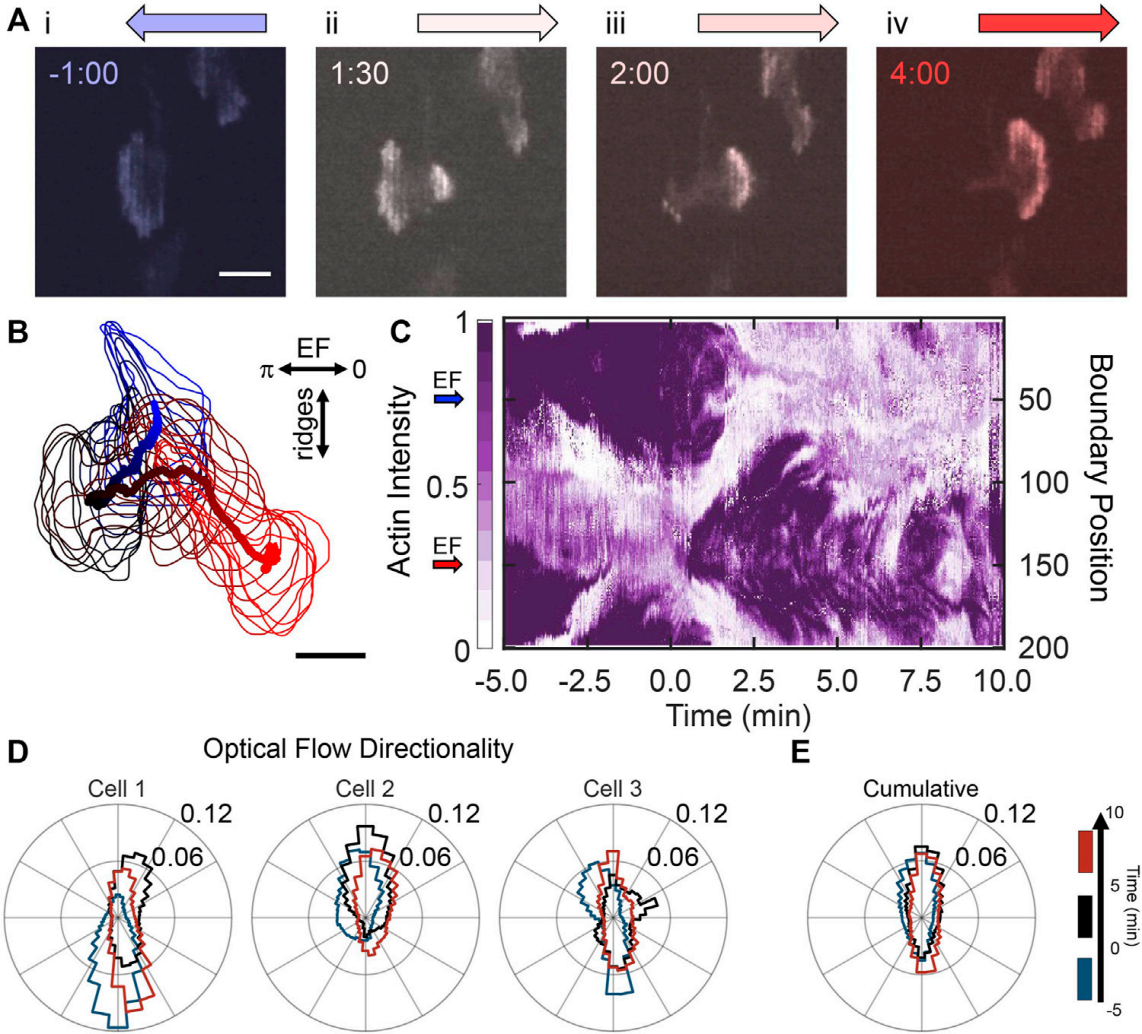
Nanoridges dominate the local guidance of actin waves in the presence of competing EF guidance. **(A)** A YFP-actin-labelled, differentiated HL60 cell on nanoridges aligned perpendicularly to the EF. The cathodal direction is to the left at negative times; the EF reversal occurred at time zero. **(B)** Path of a cell centroid with boundary shape from −5 to 10 min, plotted every 0.5 min. **(C)** Evolution of normalized boundary actin fluorescence intensity. **(D)** Three characteristic examples of individual cell normalized distributions (in counts) of optical-flow directions on perpendicular nanoridges, shown for the time ranges: (blue) 5 min before the field was reversed; (black) 5 min after the field was reversed; and (red) the period 5–10 min after the field was reversed, leftmost distribution for cell in **(A–C)**. **(E)** The cumulative flow-direction distribution for the same time intervals for all cells (n = 9).

Next, we oriented the nanoridges perpendicular to the EF to investigate the response of actin dynamics to competing guidance. Figure 3A shows actin-fluorescence images of a representative cell before and after EF reversal. Before EF reversal, the cell was oriented with its front towards the cathode (Figure 3Ai). After EF reversal (Figure 3Aii), new actin waves nucleated on the side of the cell facing the new cathode and were isolated from the previous actin front. This dynamic behavior bears resemblance to the independent actin patches in Figure 1D. Shortly after EF reversal, the emerging actin waves formed a new actin front (Figure 3Aiii–iv, Supplementary Movie S4). The actin’s response to the EF reversal is evident from the actin activity around the boundary of the cell (Figure 3B). A kymograph of the cell boundary’s fluorescence intensity in (Figure 3C) shows a jump in actin intensity on the boundary from left (boundary position 50) to right (boundary position 150) shortly after the EF reversal. These results suggest that switching the EF biases the locations at which actin waves nucleate and, in combination with nanoridges, promotes actin-rich regions that emerge independently from the cell polarity. That is, new nucleation locations may arise far from the active cell front in previously inactive regions on the cell membrane.

Although actin polymerization occurs on the side of the cell closest to the cathode, the nanoridges had a significant effect on the local dynamics of the actin. The strong preference of actin waves to conform to the nanoridges is seen in the actin-flow distributions for three representative cells and the cumulative flow distributions in Figures 3D,E, respectively. Again, the blue, black, and red curves correspond to before EF switching, the 5 min after switching, and the following 5 min, respectively (see Supplementary Figure S2 for similar quantification for cells responding to the initial application of an EF). For all cells, there is strong alignment of the actin flow with the nanoridges. These data indicate that the EF reversal provides a slight preference for flow in the direction of the new EF.

**FIGURE 4:**
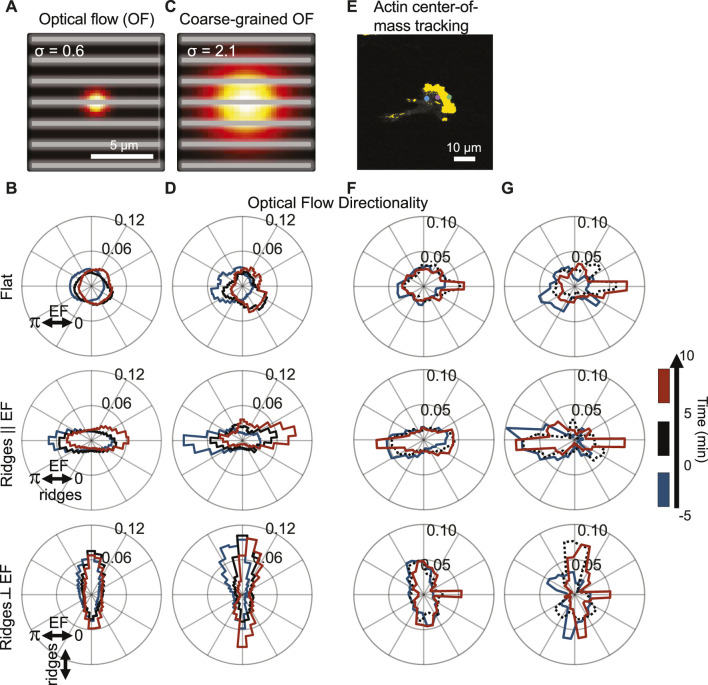
Actin dynamics analyzed on multiple scales with cooperating and competing nanotopography and EF guidance; actin polymerization is predominantly guided by nanotopography, but the guidance is biased by the EF. **(A)** Visualization of spatial scale used for optical-flow calculations (gray lines = ridges). **(B)** Cumulative flow-direction distribution shown for all cells on (top) a flat substrate, (middle) nanoridges parallel to the EF, and (bottom) nanoridges perpendicular to the EF for the three time intervals shown on the right. **(C)** Visualization of the spatial scale used for coarse-grained optical-flow calculations to capture larger-scale actin waves. **(D)** Cumulative flow-direction distribution shown for the same time intervals as **(B)** for all substrate conditions. **(E)** Visualization of actin center-of-mass tracking via the Crocker-Grier algorithm (Crocker and Grier, 1996), with track results from three time points shown. Actin centroid tracking for all substrate conditions with **(F)** *dt* = 2 s and **(G)** *dt* = 120 s for the same time intervals as **(B,D)**.

The widths of individual actin waves on nanoridges are less than 1 μm, whereas the polymerizing front in a migrating cell covers the entire cell width (∼10 µm). Accordingly, we compared the actin-flow measurements on different scales (Figure 4A). We contrast the actin dynamics from Figures 2, 3 on the 0.6 µm scale (Figure 4B) to the dynamics over a length scale of 2.1 µm (Figure 4C), which is larger than the ridge spacing of 1.5 µm. Guidance by the perpendicular EF is clear on the longer length scale. The bias between the red and blue distributions is a further indication that the actin waves respond to a change in the EF globally, whereas local actin waves predominantly follow the nanoridges. Next, we quantified the actin dynamics for different time scales (Figures 4F,G). We used a Crocker-Grier-based code (Crocker and Grier, 1996) to determine the motion of the actin fronts for a time window of *dt* = 2 s (Figure 4F) and 120 s (Figure 4G). The actin fronts follow the changing electric field more strongly over the longer time scale than over the shorter time scale.

**FIGURE 5:**
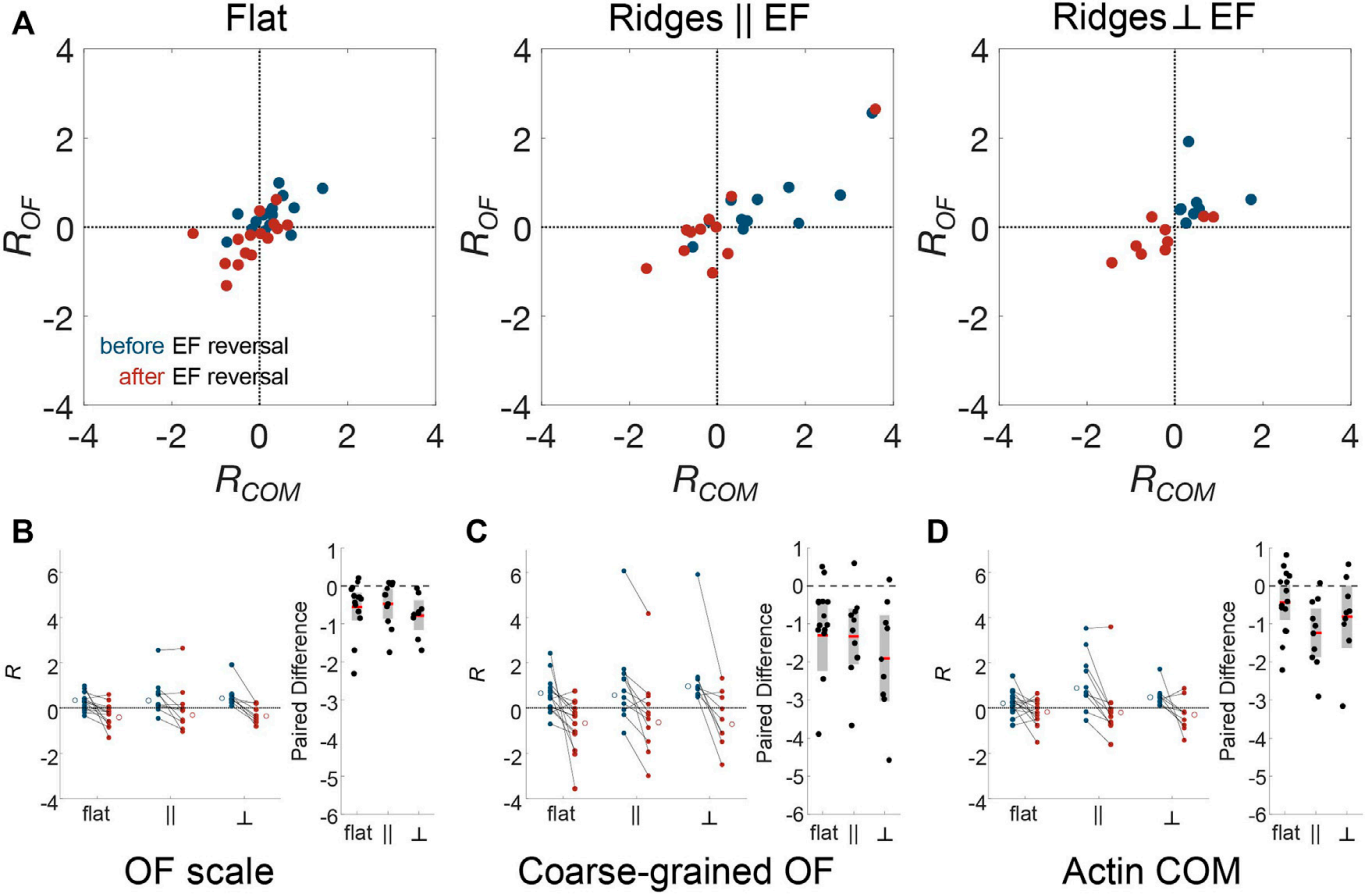
Actin dynamics on multiple scales is correlated, and responds to changing EF direction, independent of nanotopographic guidance. To compare EF guidance of actin waves and, therefore, the cell polarity across substrate type and analysis method, the angular distributions were split by angle, and the logarithm of the ratio of leftward angle counts to rightward angle counts (*R*) was calculated. Positive *R* indicates flow towards the left and negative *R* indicates flow towards the right. **(A)** Comparison between the small-scale optical-flow measurement (Figures 4A,B) and the whole-cell, actin-center-of-mass measurement (Figures 4E,F) before (blue) and after (red) EF reversal. Combined results of cell-scale polarity before and after EF reversal at **(B)** optical-flow scale, **(C)** the coarse-grained OF scale, **(D)** the cellular scale, and their paired differences, respectively.

Because there is significant cell-to-cell variability, we further assessed whether dynamics is correlated across cells by calculating changes in the logarithmic ratio of leftward to rightward flow dynamics of actin, which we denote *R*. Negative and positive *R* values correspond to left and right bias in actin flow, respectively. Thus, a change in *R* provides a sensitive indication of the existence of alignment to the external cues. As above, we compared the actin dynamics in the period of 5–10 min after the EF reversal to those in the 5 min immediately prior to the reversal. Figure 5A is a scatter plot of *R* using the actin-intensity tracks in Figure 4F (*x*-axis) and the optical-flow measurements plotted in Figure 4B (*y*-axis). This plot highlights the significant cell-to-cell variability. Here, each point corresponds to data from a separate experiment. A trend in all the datasets is that the global actin intensity dynamics and the local actin flow are correlated for all three guidance conditions. The strongest correlation across spatial scales is observed for nanoridges oriented parallel to the EF. The strong correlation across scales implies that the degree of guidance is comparable on both submicron and cellular scales.

Note that some cells do not follow the directional guidance of the EF. Specifically, *R* is negative for blue circles and positive for red circles. To assess whether these cells are still guided by EFs, we used changes in *R* to quantify the likelihood that EF reversal affects individual cells. Figures 5B–D show the change in *R* for optical flow, coarse-grained optical flow, and global actin intensity for the three experimental conditions. Analysis of the paired difference shows that both at the OF scale (Figure 5B) and the coarse-grained scale (Figure 5C), the actin dynamics changed in a manner that is consistent with the changing direction of the EF. The cell-scale actin dynamics (Figure 5D), for which there are fewer data points due to the nature of the measurements, shows a significant switch only on nanoridges parallel to the EF. Indeed, on the OF scale, even though the actin dynamics did not always point preferentially in the direction of the cathode, the actin polymerization was drawn toward the new cathode for all cells. This observation suggests that actin responds to changes in the EF direction, and not just to an ambient EF.

## Discussion

Here we investigated actin polymerization in the presence of two distinct guidance cues: nanotopography and EFs. As seen in Figure 1, actin polymerization occurs predominantly at the edge of the cell, driving local protrusions. On flat substrates, a single actin-rich lamellipodium typically covers the entire leading edge, resulting in polarization and directed migration across applied guidance cues (Inagaki and Katsuno, 2017). With the addition of nanoridges (esotaxis), actin polymerization decouples from the single lamellipodium and can nucleate at locations that are independent of cell polarity. These locations form one-dimensional actin waves that propagate along the direction of the nanoridges (Sun et al., 2015) and are biased in the direction the external EF (electrotaxis).

Individual actin waves do not reverse direction. Instead, on flat substrates the waves execute broad turns that avoid recently polymerized regions, resulting in a traditional U-turn phenotype. In contrast, on nanoridges pre-existing one-dimensional waves cannot turn, so the response to the reversing EF involves new actin polymerization at locations distinct from the original wave front. On a cellular scale, these multiple new actin waves can recapitulate the familiar U-turn phenotype, resulting in the cell changing migration directions.

Our optical-flow results show that in an EF, actin waves are biased toward the cathode (Figures 2, 3), with stronger guidance when the EF direction is switched than for the initial appearance of an EF. Multiscale analysis (Figures 4, 5) reveals that the preferred direction of actin waves is along the direction of nanoridges, whether perpendicular or parallel to the EF. For both nanoridge orientations, the preferred direction is biased slightly toward the cathode. Although cells migrate preferentially toward the cathode on average, some cells move preferentially toward the anode both before and after the switch in cathode directions. Such behavior is most common for cells on flat resin, for which motion is dominated by a cell’s intrinsic polarity, leading to directed migration that can persist for many minutes before adaptation to external cues. However, even when the dynamics of a cell is dominated by cell polarity, EFs preferentially guide actin dynamics toward the cathode (Figures 5B–D). Switching the EF always generates a stronger response towards the new cathode, regardless of initial migration direction.

Our work reveals the mechanism through which actin waves can integrate multiple physical guidance cues. Nanotopography acts at a submicron scale, resulting in cell-wide, one-dimensional waves that are guided along ridges individually. EFs act on the cellular scale, biasing the direction of actin waves, and therefore cell migration, toward the cathode. When cells are exposed to competing cues, the actin dynamics predominantly follows the nanotopography. Nevertheless, EFs dominate cell guidance in the long term by biasing the locations of new actin waves and providing a unidirectional guidance cue for the waves, in contrast to the bidirectional guidance cue of the nanoridges.

Thus, nanotopography, as a local guidance cue, impacts guided cell migration in two ways. First, nanoridges that are aligned parallel to the guidance cue reduce the two-dimensional guidance problem to a one-dimensional guidance problem, thus making it easier to detect weak, unidirectional guidance cues. Second, nanoridges that are aligned perpendicular to the guidance cue can provide strong local steering without significantly diminishing the effectiveness of the unidirectional guidance cue. Therefore, our work has implications for real wound sites, which include not only EFs, but also multiple chemotactic signals that act on cellular scales (Liu et al., 2021; Moarefian et al., 2021; Yao et al., 2022). Our results indicate that when nanotopography is the only local guidance cue, cells can respond to this texture while still moving toward the site of a wound. The characteristics of actin waves thus offer a powerful organizing principle for understanding the cell behavior in complex microenvironments.

## Data Availability

The raw data supporting the conclusion of this article will be made available by the authors, without undue reservation.
