# Supplementary material for: Actin Dynamics as a Multiscale Integrator of Cellular Guidance Cues
Source: Front Cell Dev Biol. 2022 Apr 27;10:873567. doi: 10.3389/fcell.2022.873567 (PMC9092214; doi:10.3389/fcell.2022.873567)
Supplement: Supplementary file 2 [file Table1.DOCX]

Supplementary Material

# Supplementary Figures


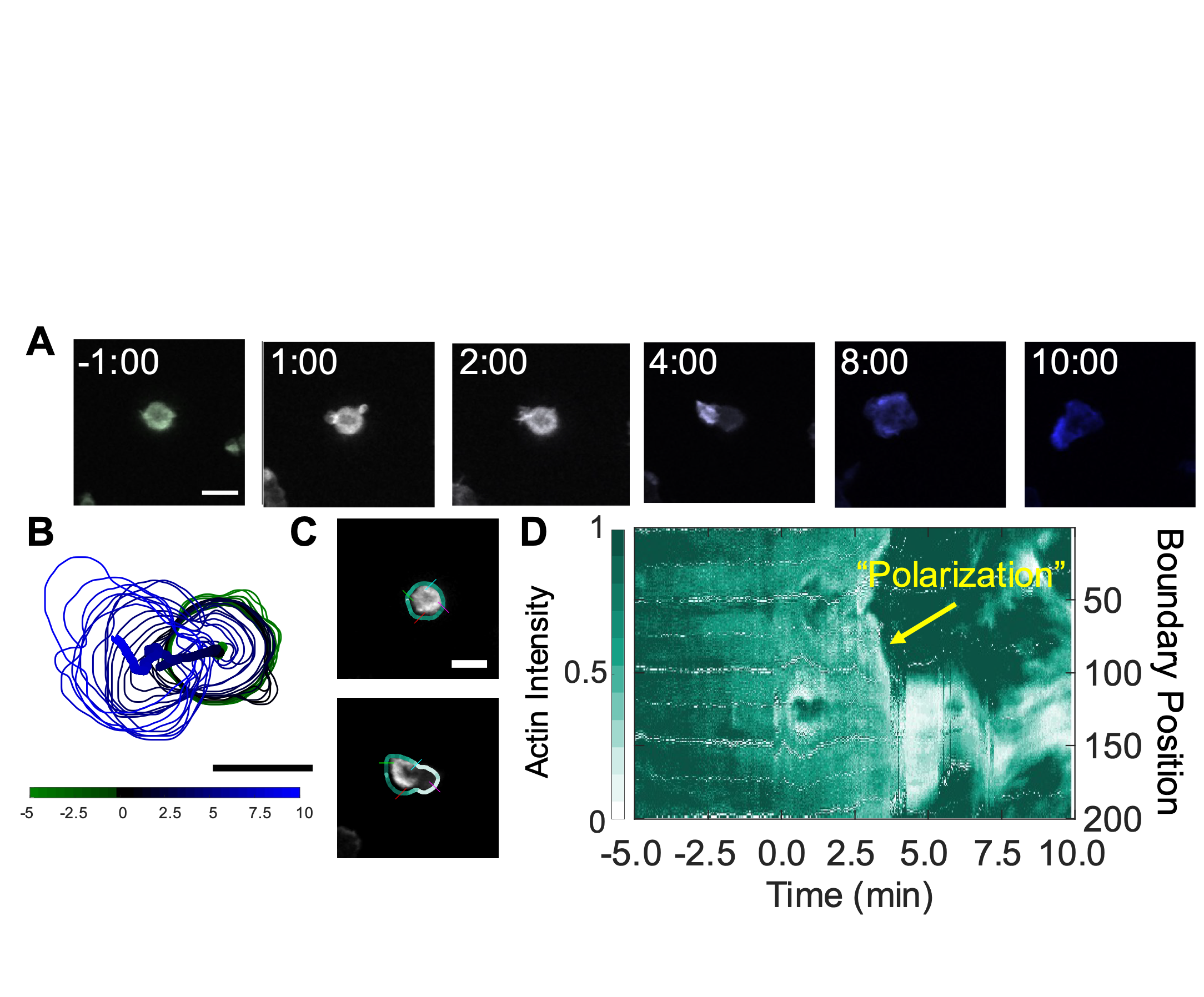


**Supplementary Figure 1.** Activation of HL60 cell by introduction of EF. (A) Time-lapse snapshots (in minutes) of differentiated YFP-actin labelled HL60 cell on a flat substrate. No field in negative time. EF cathodal direction is to the left in positive time. Scale bars are 10 µm. (B) Boundary shape and path of the centroid of the cell from (A) from -5:00 min to 10:00 min in 0.5-min increments, with (C) an example of the extracted shape of the cell colored by actin intensity. (D) Evolution of the normalized boundary actin fluorescence intensity visualized as a kymograph with the polarization/elongation even labeled in yellow.


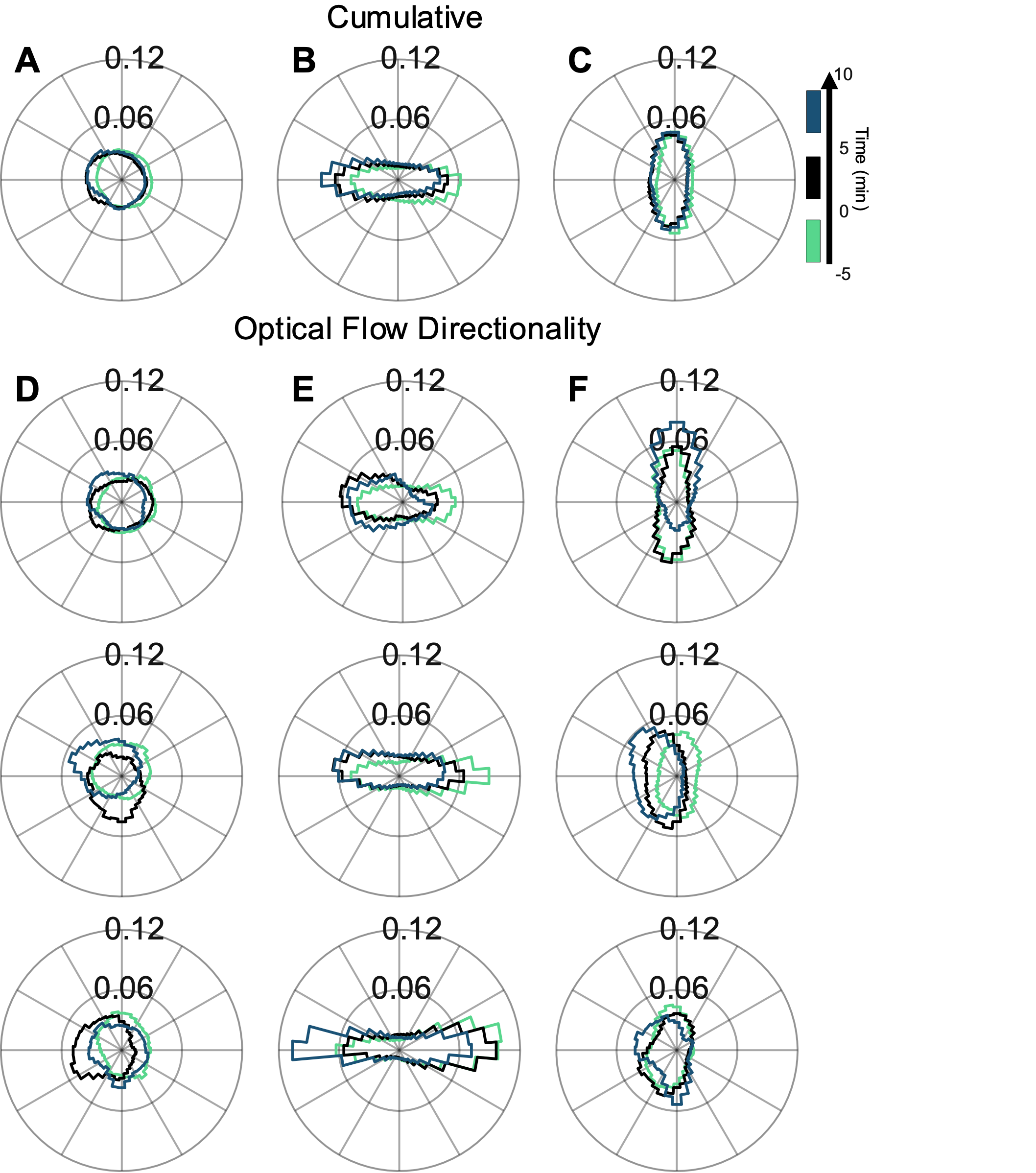


**Supplementary Figure 2.** Actin dynamics analyzed with the initial EF stimulation. (A) Cumulative flow-direction distribution shown for the time ranges: (green) 5 min before EF stimulation; (black) 5 min after field was initiated; and (blue) the period 5 min to 10 min after the field was initiated for all cells on (left) flat substrate, (middle) nanoridges parallel to the EF, and (right) nanoridges perpendicular to the EF. Three characteristic examples of individual, cell-normalized distributions (in counts) of optical-flow directions on the (D) flat substrate, (E) nanoridges parallel and (F) nanoridges perpendicular to the EF.


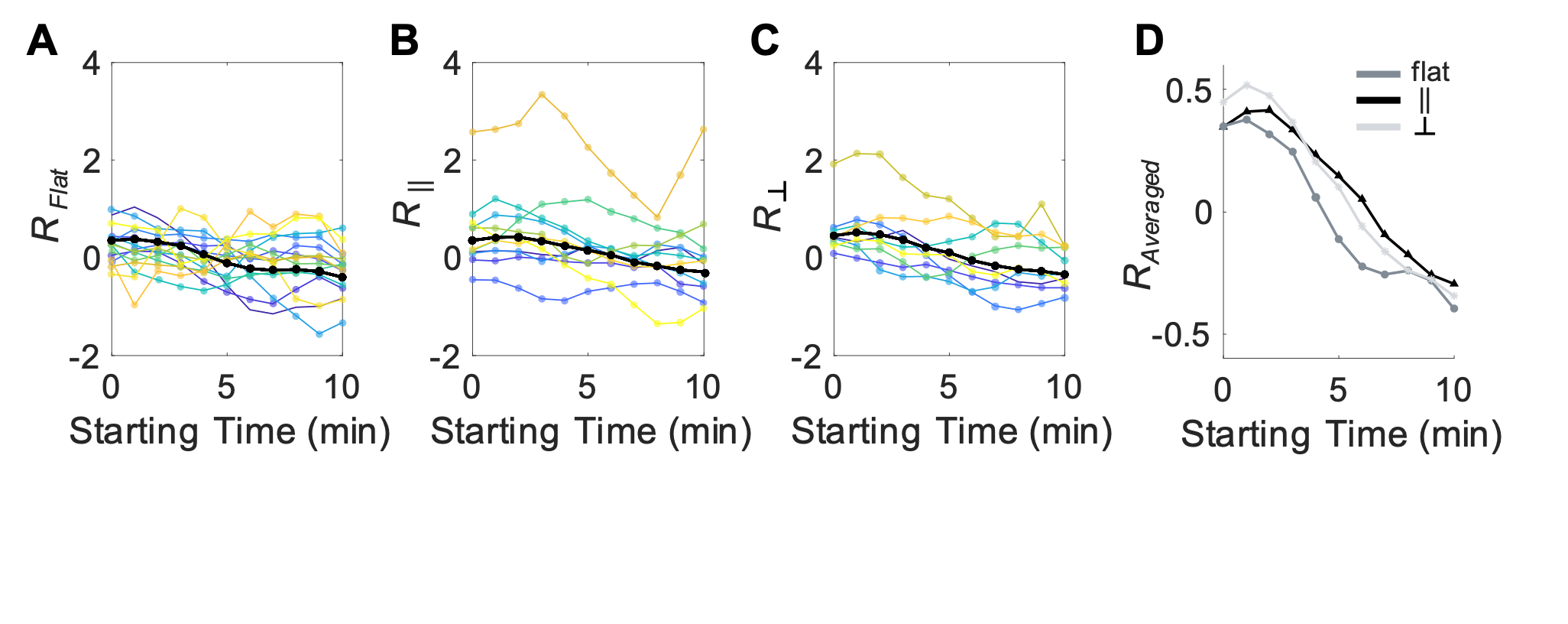


**Supplementary Figure 3.** Logarithm of the ratio of leftward angle counts to rightward angle counts (*R*) calculated across 5-min intervals and displayed at each minute for each experiment (averaged with black line) for (A) flat substrate, (B) nanoridges parallel and (C) nanoridges perpendicular to the EF. (D) Averaged *R* for the same three conditions.

Supplementary Movie 1.

A YFP-actin-labelled, neutrophil-like HL60 cell on a flat substrate. The EF direction is reversed at *t=0*. The scale bar is 10 μm.

Supplementary Movie 2.

A YFP-actin-labelled, neutrophil-like HL60 cell on nanoridges parallel to the EF axis. The EF direction is reversed at *t = 0*. The scale bar is 10 μm.

Supplementary Movie 3.

A YFP-actin-labelled, neutrophil-like HL60 cell on flat substrate. The EF is introduced at *t = 0*. Scale bar 10 μm.

Supplementary Movie 4.

A YFP-actin-labelled, neutrophil-like HL60 cell on nanoridges perpendicular to the EF axis. The EF direction is reversed at *t = 0.* The scale bar is 10 μm.
